# Structural studies of the periplasmic portion of the diguanylate cyclase CdgH from *Vibrio cholerae*

**DOI:** 10.1038/s41598-017-01989-6

**Published:** 2017-05-12

**Authors:** Min Xu, Yi-Zhi Wang, Xiu-An Yang, Tao Jiang, Wei Xie

**Affiliations:** 1 National Laboratory of Biomacromolecules, CAS Center for Excellence in Biomacromolecules, Institute of Biophysics, Chinese Academy of Sciences, Beijing, China; 2 University of Chinese Academy of Sciences, Beijing, China; 3 School of Life Sciences, Tianjin University, Tianjin, China

## Abstract

Cyclic diguanylate monophosphate (c-di-GMP) is a second messenger involved in bacterial signal transduction and produced by diguanylate cyclases (DGCs) generally containing highly variable periplasmic signal-recognition domains. CdgH is a DGC enzyme that regulates rugosity associated phenotypes in *Vibrio cholerae*. CdgH has two N-terminal tandem periplasmic substrate-binding (PBPb) domains for its signal recognition; however, the role of the tandem PBPb domains remains unclear. Here, we reported the crystal structure of the periplasmic portion of CdgH, which indicated that both tandem PBPb domains consist of typical interlobe ligand-binding architecture. Unexpectedly, the PBPb-I domain binds an L-arginine which apparently has been co-purified from the *E. coli* expression system, whereas the PBPb-II domain is in an unliganded open state. Structural comparison with other amino acid-binding proteins indicated that despite similar ligand-binding pockets, the PBPb-I domain possesses two ligand-binding residues (E122 and Y148) not conserved in homologs and involved in hydrophilic and hydrophobic interactions with L-arginine. Isothermal titration calorimetry indicated that the PBPb-I is primarily an L-arginine/L-lysine/L-ornithine-binding domain, whereas the PBPb-II domain exhibits a preference for L-glutamine and L-histidine. Remarkably, we found that the periplasmic portion of CdgH forms a stable dimer in solution and L-arginine binding would cause conformational changes of the dimer.

## Introduction

Cholera is a severe diarrheal disease caused by the Gram-negative bacterium *Vibrio cholerae* and is responsible for the infection of 3 to 5 million individuals and 100,000 to 120,000 deaths annually^1^. In the aquatic phase of its life cycle, *V. cholerae* is a planktonic, free-swimming organism, but prefers to form a sessile biofilm on the chitin surfaces of aquatic organisms^2–4^. The biofilm matrix aids the survival of *V. cholerae* by enabling it to overcome nutrient limitations, as well as providing protection from other environmental stressors^5^. After entering a human host along with contaminated water or food, *V. cholerae* responds to changes in environmental conditions by transiting from an aquatic bacterium to a human pathogen and producing a toxin-co-regulated pilus and cholera toxin that cause host illness^6, 7^. The responses of *V. cholerae* to environmental signals involve surface attachment, biofilm formation, orbiting and roaming motility, virulence-gene expression, and life cycle progression^5^.

3′,5′-cyclic diguanylate monophosphate (c-di-GMP) is a ubiquitous secondary messenger that plays a key role in response regulation and lifestyle conventions of pathogenic bacteria, including *V. cholerae* ^8–12^. Increases in c-di-GMP levels induce increased expression of various factors necessary for the establishment and maintenance of biofilm communities, whereas decreased levels usually lead to enhanced expression of virulence and motility factors related to biofilm degradation^5, 9, 11, 13, 14^. C-di-GMP is synthesized from guanosine triphosphate by diguanylate cyclase (DGC) enzymes and hydrolyzed to 5′-phosphoguanylyl-(3′ → 5′)-guanosine by c-di-GMP-specific phosphodiesterases (PDEs)^15, 16^. Proteins with a conserved C-terminal GGDEF domain act as DGCs^17, 18^, whereas proteins containing EAL or HD-GYP domains act as PDEs^8, 18, 19^. The *V. cholerae* genome typically encodes 31 proteins with a GGDEF domain^10^. Interestingly, most DGCs are not only the producers of c-di-GMP but also sensors of environmental signals. In addition to the conserved C-terminal GGDEF domain, these DGCs also contain highly variable N-terminal conserved signal-recognition domains^15, 20^. The significant number of different DGCs implies that each DGC can sense and respond to specific environmental signals and thereby produce c-di-GMP as a second messenger^5^. However, little is known about the molecular mechanisms associated with this possible activity, and the functions of the variable N-terminal signal-recognition domains in most DGC enzymes remain unidentified.

*V. cholerae* CdgH is a DGC enzyme containing the conserved C-terminal cytoplasmic GGDEF domain that allows it to engage in DGC activity *in vivo* ^10^. CdgH differs from other *V. Cholerae* DGCs by having two N-terminal tandem periplasmic substrate-binding (PBPb) domains as its signal recognition domain^21^; however, the role of these tandem PBPb domains remains unclear. As previously reported, CdgH positively regulates rugosity associated phenotypes in *V. cholerae*, and *V. cholerae* CdgH mutants form less corrugated and flatter colonies as compared with those formed by wild-type variants^10^. CdgH also plays a major role in c-di-GMP synthesis by responding to the presence of bile acids^21^. CdgH deletion reduces bile-mediated induction of c-di-GMP and biofilm formation^21^. Although CdgH regulates significant *V. cholera*-related phenotypes, the environmental signals sensed by CdgH remain unknown, and the mechanisms associated with how substrate binding triggers the DGC activity of CdgH also remain elusive.

Here, we reported the crystal structure of the periplasmic portion of *V. Cholerae* CdgH. Our structure indicated that both tandem PBPb domains of CdgH contain a typical interlobe ligand-binding structural architecture. Intriguingly, PBPb-I domain is in complex with L-arginine. Our results indicated that the PBPb-I and -II domains have different amino acid binding specificity. Moreover, the periplasmic portion of CdgH was found to form a dimer in solution which could undergo conformational changes upon L-arginine binding.

## Results

### Overall structure of the periplasmic portion of CdgH

**Figure 1:**
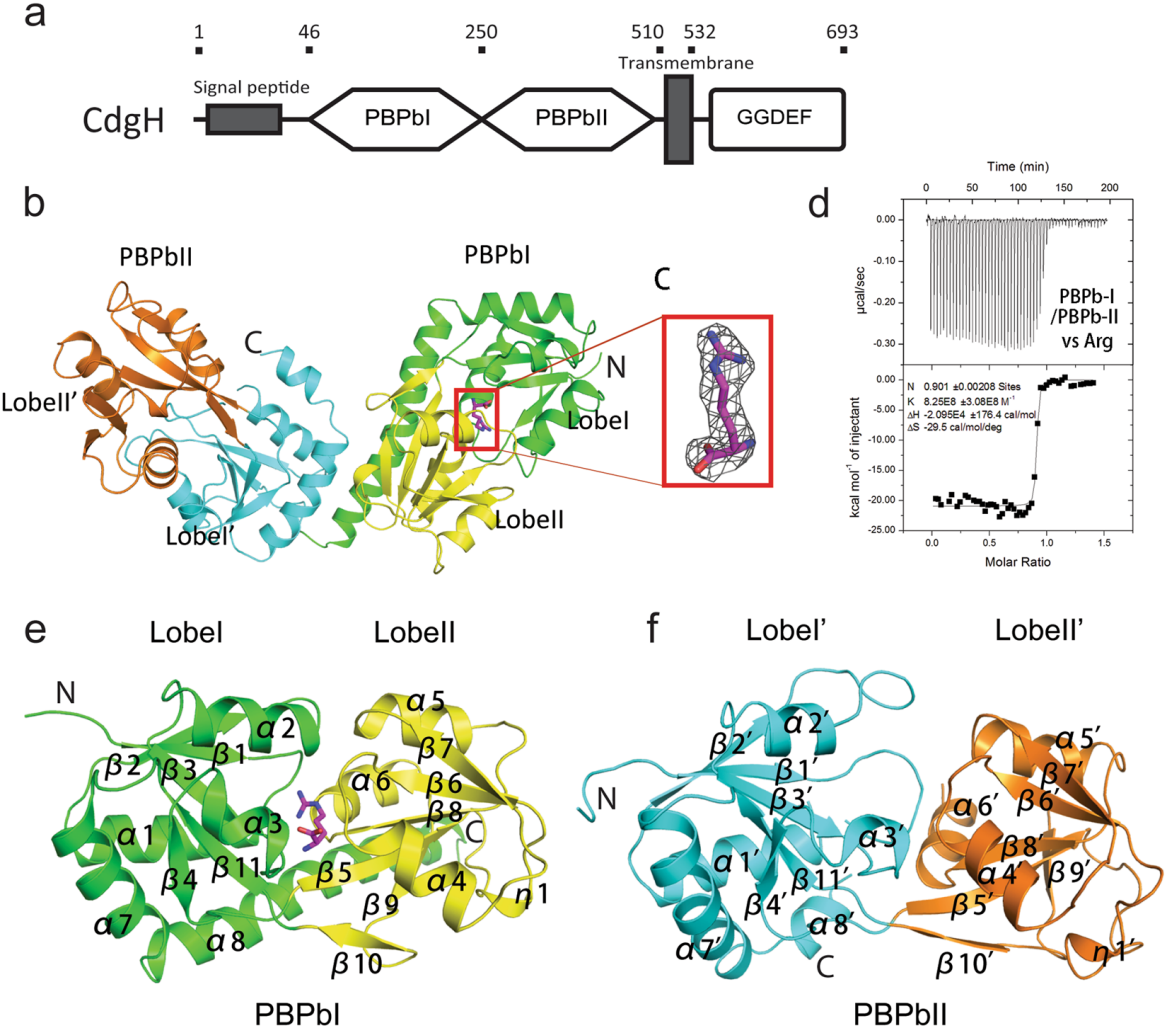
The overall structure of the periplasmic portion of CdgH. (**a**) The domain architecture of the full-length *Vibrio cholerae* CdgH protein. (**b**) The overall structure of the periplasmic portion of CdgH (46–491aa). (**c**) The electron density found in the ligand-binding pocket was contoured at 3.0 σ in the |Fo| − |Fc| maps. The L-arginine fits well in the electron density omit map. (**d**) Data of the ITC experiment involving the refolded periplasmic portion of CdgH with L-arginine. (**e**,**f**) The similar folding topologies of the PBPb-I and -II domains. Lobe-I and lobe-II of the PBPb-I domain are shown in green and yellow, while lobe-I′ and lobe-II′ of the PBPb-II domain are shown in cyan and orange. The L-arginine bound in the ligand-binding pocket is shown in magenta.

We crystallized the periplasmic portion (residues 46–491, which includes the tandem PBPb-I and PBPb-II domains) of CdgH containing a C-terminal His-tag (Fig. 1a), and solved the structure to 2.6-Å resolution using selenomethionine-substituted protein crystals by single-wavelength anomalous diffraction (SAD) method. The crystal belongs to the P6_3_22 space group, with one molecule in the asymmetric unit. Most residues (48–283, 291–437, and 443–491) were well traced in the final model (Table 1).

**Table 1:** Data-collection, phasing and refinement statistics.

Data collection	CdgH
Space group	P 6_3_ 2 2
Wavelength (Å)	0.97848
Resolution (Å)^a^	36.56–2.601 (2.694–2.601)
Cell dimensions	
a, b, c (Å)	111.71, 111.71, 138.65
α, β, γ (^°^)	90, 90, 120
Unique reflections	16125 (1554)
I/σI	28.9 (4.14)
Completeness (%)	99.0 (99.0)
R_merge_ (%)	6.3 (46.1)
Wilson B factor (Å^2^)	39.2
Refinement	
R_work_ (%)	22.92 (29.33)
R_free_(%)	28.85 (41.10)
Average B factors (Å^2^)	56.29
Root mean square deviations	
Bond lengths (Å)	0.011
Bond angles (^°^)	1.39
Ramachandran plot	
Most favored (%)	91
Additionally allowed (%)	7
Generously allowed (%)	0
Disallowed	1.9

Note: Numbers in parentheses are for the highest resolution shell.

The tandem CdgH PBPb-I and -II domains share ~19% amino acid sequence identity^22, 23^ (Fig. S1), and the crystal structure also indicated that the two domains contain similar folding topology (Fig. 1b). Both PBPb domains contain two lobes. In lobe-I and lobe-I′, the central β-sheet (β2-β1-β3-β11-β4, with β11 antiparallel to the rest) is surrounded by five peripheral α-helices (α1-α2-α3-α7-α8), whereas in lobe-II and lobe-II′, the β-sheet (β7-β6-β8-β5-β9-β10, with β5 antiparallel to the rest) is surrounded by three peripheral α-helices (α4-α5-α6). The two lobes in each domain are connected by two loop segments (β4-β5 and β10-β11) that appear to function as flexible hinge regions. Additionally, the two tandem PBPb domains are stably connected through the packing of the C-terminal portion of the α8 helix in lobe-I of the PBPb-I domain with the α7′ helix in lobe-I′ of the PBPb-II domain (Fig. 1e and f), placing the two domains perpendicular to each other.

According to the structural homology searches using the DALI server^12^, the highest scoring result for the tandem PBPb domains of CdgH is the virulence sensor-kinase BvgS^24^, the only other reported structure containing tandem periplasmic domains. BvgS serves as a prototypical two-component systems sensor-kinase in Bordetella pertussis with tandem periplasmic Venus flytrap (VFT) domains. Structural superposition revealed that although the PBPb domains of CdgH and the VFT domains of BvgS are both typically bi-lobed domains, the orientations and arrangements of the tandem periplasmic domains of the two proteins are obviously different (Fig. S2), lie in that the two PBPb domains in CdgH are stably connected by a unique long α8 helix, while the tandem VFT domains of BvgS are loosely connected by a flexible loop, indicating a novel tandem PBPbs structure in CdgH.

Meanwhile, DALI searches also revealed that the highest scoring result for the PBPb-I domain was the ancestral arginine-binding protein AncQR^25^ [Z-score: 18.4; root mean square deviation (RMSD): 3.1], and the PBPb-II domain was the histidine kinase sensor domain of HK29S^26^ (Z-score: 21.3; RMSD: 2.6), indicating that both CdgH PBPb domains contained a typical interlobe ligand-binding architecture, despite sharing relatively low sequence identities.

Interestingly, the electron density omit map indicated the presence of additional density positions in a deep cleft formed by the two lobes of the PBPb-I domain (Fig. 1c). The length and shape of the density correspond to an L-arginine molecule, and isothermal titration calorimetry (ITC) experiments confirmed a very strong binding affinity of L-arginine with the tandem PBPb domains (K_d_ = 1.21 × 10^−9^ M; Fig. 1d and Table 2). The actual affinity may be stronger than this calculated affinity, because the steep ITC binding curve makes robust fitting difficult. The affinity of L-arginine with the tandem PBPb domains is much stronger than that with other L-arginine binding proteins, such as LAOBP (K_d_ = 14 × 10^−9^ M)^27^. This unexpected bound L-arginine may be co-purified from the *E. coli* expression system. The interlobe ligand-binding pocket agrees with many other reported bacterial ligand-binding structures. However, we observed no similar density in the PBPb-II domain, indicating that the PBPb-I domain of our structure is in a ligand-binding conformation, and the PBPb-II domain is in a ligand-free conformation (Fig. 1e and f).

**Table 2:** ITC analysis of interactions between different amino acids and the periplasmic portion of CdgH with different truncations and mutants.

Protein	Ligand	n	K_a_ (M^−1^)	K_d_ (M)	ΔH (kcal/mol)
PBPb-I /PBPb-II	L-Arg	0.90	8.25E8 ± 3.08E8	1.21E-9	−20.98 ± 0.17
PBPb-I		1.07	2.77E7 ± 2.96E6	3.61E-8	−12.87 ± 0.04
PBPb-I^E56A^		0.86	5.37E5 ± 1.94E4	1.86E-6	−10.62 ± 0.06
PBPb-I^R109A^		0.76	1.14E5 ± 6.33E3	8.77E-6	−15.90 ± 0.31
PBPb-I^E122A^		0.81	3.42E4 ± 1.34E3	2.92E-5	−10.49 ± 0.29
PBPb-I^E56A/R109A/E122A^		NB	NB	NB	NB
PBPb-I^E56A/R109A/E122A^/PBPb-II		1.00	7.19E2 ± 1.60E2	1.39E-3	−0.89 ± 0.11
PBPb-I	L-Lys	1.26	1.50E6 ± 1.58E5	6.67E-7	−6.02 ± 0.05
PBPb-I^E56A^		0.97	1.21E5 ± 4.90E3	8.26E-6	−10.85 ± 0.12
PBPb-I^R109A^		1.00	2.49E3 ± 1.61E2	4.01E-4	−5.66 ± 0.24
PBPb-I^E122A^		1.00	5.77E3 ± 1.91E2	1.73E-4	−4.60 ± 0.08
PBPb-I^E56A/R109A/E122A^		NB	NB	NB	NB
PBPb-I^E56A/R109A/E122A^/PBPb-II		1.00	4.43E2 ± 0.42E2	2.26E-3	−12.14 ± 0.75
PBPb-I	L-Orn	1.31	2.40E5 ± 3.37E4	4.17E-6	−3.61 ± 0.08
PBPb-I^E56A^		1.10	4.11E4 ± 8.02E3	3.17E-5	−6.08 ± 0.55
PBPb-I^R109A^		1.00	6.53E2 ± 1.17E2	1.53E-3	−5.09 ± 0.75
PBPb-I^E122A^		1.00	4.41E2 ± 0.98E2	2.27E-3	−9.74 ± 1.88
PBPb-I^E56A/R109A/E122A^		NB	NB	NB	NB
PBPb-I^E56A/R109A/E122A^/PBPb-II		1.00	3.14E2 ± 0.40E2	3.18E-3	−2.35 ± 0.22
PBPb-I	L-Gln	1.51	1.48E4 ± 6.49E3	6.67E-5	−3.57 ± 1.02
PBPb-I^E56A^		0.74	1.07E4 ± 4.49E3	9.34E-5	−7.49 ± 5.40
PBPb-I^R109A^		NB	NB	NB	NB
PBPb-I^E122A^		NB	NB	NB	NB
PBPb-I^E56A/R109A/E122A^		NB	NB	NB	NB
PBPb-I^E56A/R109A/E122A^/PBPb-II		1.23	1.26E4 ± 1.40E3	7.94E-5	−3.22 ± 0.12
PBPb-I	L-His	1.25	4.31E4 ± 3.67E3	2.32E-5	−7.90 ± 0.28
PBPb-I^E56A^		0.75	1.04E4 ± 5.05E3	9.62E-5	−11.16 ± 9.32
PBPb-I^R109A^		1.00	0.53E2 ± 0.06E2	1.90E-2	−5.04 ± 0.57
PBPb-I^E122A^		1.00	3.23E2 ± 0.12E2	3.09E-3	−2.49 ± 0.07
PBPb-I^E56A/R109A/E122A^		NB	NB	NB	NB
PBPb-I^E56A/R109A/E122A^/PBPb-II		1.31	1.08E4 ± 1.58E3	9.25E-5	−1.51 ± 0.10

NB: no detactable binding.

In addition, it has been reported that CdgH activity can be induced by bile acids *in vivo*, however, it maintains robust activity upon exogenous expression even in the absence of bile acids, suggesting that CdgH is controlled indirectly by bile through sensing perturbations in the membrane^21^. To elucidate whether the tandem PBPb domains of CdgH can bind bile acids, an ITC assay was performed and no measurable binding affinity was detected between the tandem PBPb domains of CdgH and bile acids. This result, in line with the previous report, suggests that bile acids induce CdgH activity by other ways rather than by binding to the periplasmic portion of CdgH.

### Structural details of the ligand-binding site of the PBPb-I domain

**Figure 2:**
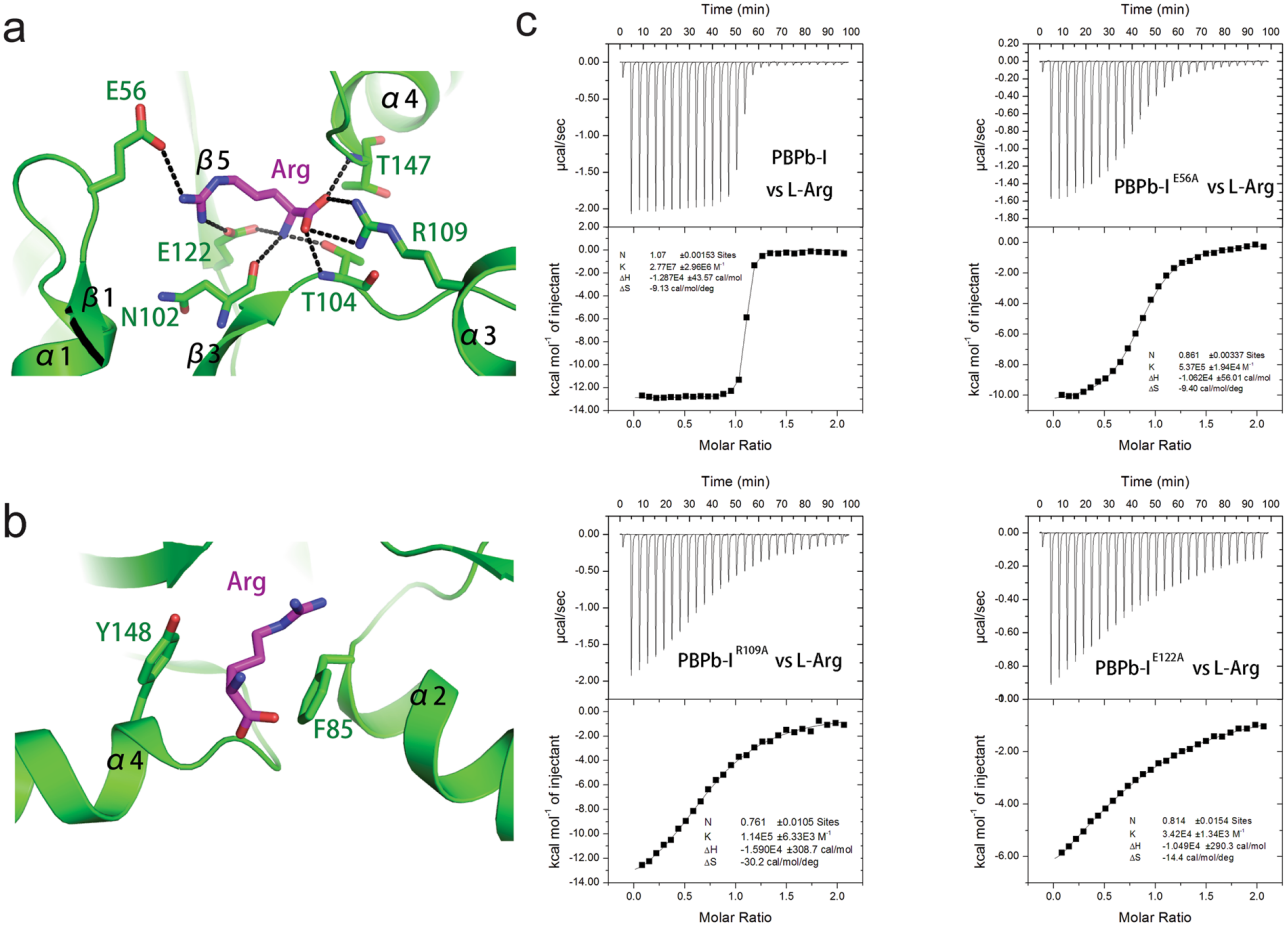
Structural details of the ligand-binding pocket in the PBPb-I domain. (**a**) Hydrophilic interactions. (**b**) Hydrophobic interactions. The PBPb-I domain is shown as a green cartoon, and the residues involved in ligand-binding interactions are shown as green sticks. L-arginine is shown in magenta. (**c**) Data from ITC experiments involving the PBPb-I domain (the refolded PBPb-I or PBPb-I mutants, as indicated) in complex with L-arginine.

In the PBPb-I domain bound with L-arginine, the key residues involved in hydrophilic interactions with L-arginine are E56 on the β1-α1 loop, T104 on the β3-α3 loop, R109 on the α3 helix, and E122 on the β5 strand. The α-amino group of L-arginine is triangulated by contacting with the hydroxyl group of T104, the carboxyl group of E122, and the main-chain oxygen atom of N102. The α-carboxyl group of L-arginine forms salt bridges with the R109 side chain (NH1 And NH2) and hydrogen bond interactions with the main-chain nitrogen atoms of T104 and T147. Additionally, the L-arginine side chain (NH1 And NH2) forms two salt bridges with E56 and E122 (Fig. 2a). In addition to the hydrophilic interactions, the L-arginine side chain is sandwiched by two hydrophobic aromatic rings from F85 and Y148 (Fig. 2b). These interactions promote tight ligand binding within the interlobe pocket to render a ligand-bound closed conformation.

### Comparison of the CdgH PBPb-I domain with other amino acid-binding proteins

**Figure 3:**
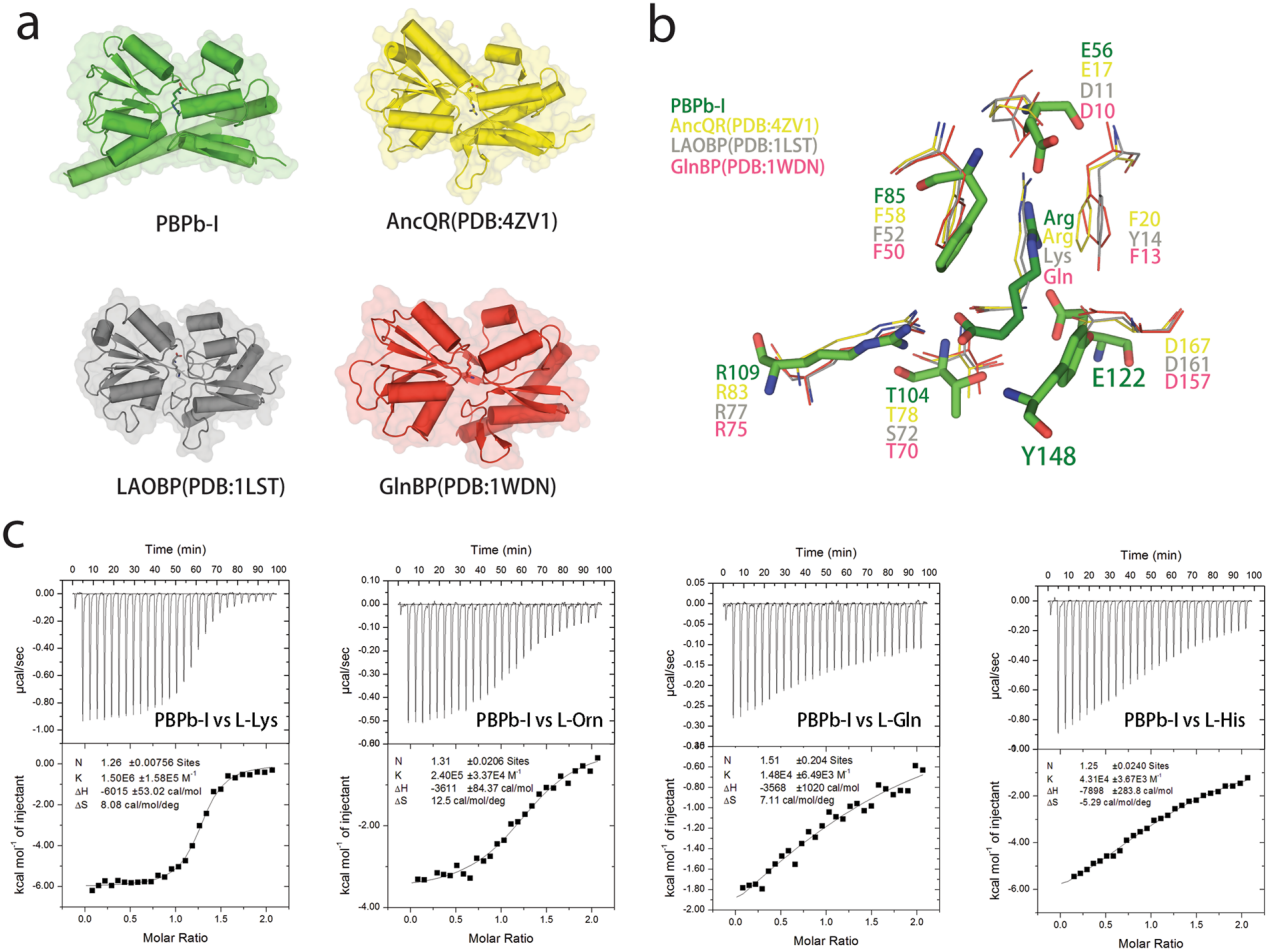
The ligand-binding properties of the PBPb-I domain. (**a**) Structural comparison between the PBPb-I domain (green) with other ligand-binding proteins. AncQR is shown in yellow, LAOBP is shown in gray, and GlnBP is shown in red. (**b**) Superposition of the key ligand-binding residues of the PBPb-I domain (green), AncQR (yellow), LAOBP (gray), and GlnBP (red). (**c**) Data from ITC experiments involving the refolded PBPb-I domain with L-lysine, L-ornithine, L-glutamine and L-histidine.

To elucidate the ligand-binding specificity of the CdgH PBPb-I domain, we compared our structure with the structural homology searching top hits from the DALI server, including AncQR^25^ (PDB: 4ZV1), a synthetical bacterial Glutamine/Arginine-binding protein that is involved in amino acid transport, LAOBP^28^ (PDB: 1LST), a bacterial periplasmic binding proteins involved in substrate transport and chemotaxis in *Salmonella typhimurium* and GlnBP^29^ (PDB: 1WDN), a component of the ligand-specific periplasmic glutamine permease system in *Escherichia coli* ^29^ (Fig. 3a). As shown in Fig. 3b, all ligand α-carboxyl and α-amino groups were stabilized by arginine side chains in each of the proteins (R109 of CdgH, R83 of AncQR, R77 of LAOBP, and R75 of GlnBP) and the hydroxyl group of threonine or serine residues (T104 of CdgH, T78 of AncQR, S72 of LAOBP, and T70 of GlnBP). Moreover, all ligand side chains were stabilized by conserved aspartate/glutamate residues (E56 of CdgH, E17 of AncQR, D11 of LAOBP, and D10 of GlnBP). In the CdgH PBPb-I domain, the α-amino group and the arginine side chain also interacted with E122, which was not observed in other complexes. Except for the CdgH PBPb-I domain, the α-amino groups of ligands in other complexes formed contacts with aspartate side chains (D167 of AncQR, D161 of LAOBP, and D157 of GlnBP) and were sandwiched by two hydrophobic residues. The hydrophobic residue F85 in the CdgH PBPb-I domain is similar in location to that of F58 from AncQR, F52 from LAOBP, and F50 from GlnBP. However, the position of the second hydrophobic residue (Y148 from CdgH) differs from those of the other ligand-binding proteins (F20 of AncQR, Y14 of LAOBP, and F13 of GlnBP). Structural comparisons and sequence alignment of the ligand-binding pockets revealed that despite similarities in ligand binding, differences in the CdgH PBPb-I domain involving E122 and Y148 involved in hydrophilic and hydrophobic interactions with L-arginine, respectively, are not conserved in the other proteins (Fig. S1). These findings suggested that the CdgH PBPb-I domain possesses a new L-arginine-binding mode.

### Mutation analysis of the CdgH PBPb-I ligand-binding pocket

To confirm the importance of the key residues involved in ligand recognition, we expressed and purified the PBPb-I domain (46–250) alone in *E.coli*, and constructed several mutants (PBPb-I^E56A^, PBPb-I^R109A^, PBPb-I^E122A^, and PBPb-I^E56A/R109A/E122A^) based on structural details of the ligand-binding site. These wild-type PBPb-I domain and mutants are all soluble by itself in solution. Then we tested their affinities for L-arginine by ITC (Fig. 2c and Table 2). The ITC results showed that the PBPb-I^E56A^ (K_d_ = 1.86 × 10^−6^ M), PBPb-I^R109A^ (K_d_ = 8.77 × 10^−6^ M), and PBPb-I^E122A^ (K_d_ = 2.92 × 10^−5^ M) mutants resulted in 51.5-, 242.9-, and 808.9-fold reductions in binding affinity, respectively, relative to the wild-type PBPb-I domain (K_d_ = 3.61 × 10^−8^ M). Moreover, the PBPb-I^E56A/R109A/E122A^ triple mutant led to no measurable binding ability, implying the essential functions of these three residues for recognizing L-arginine.

### The ligand-binding properties of the CdgH PBPb-I domain

To determine the ability of the CdgH PBPb-I domain to bind a broad range of ligands in addition to L-arginine, we employed ITC to test the binding affinities of the wild-type PBPb-I domain with various amino acids (L-lysine, L-ornithine, L-glutamine, L-histidine, and L-aspartate) (Fig. 3c and Table 2). The results showed that the highest binding affinity was associated with L-lysine (K_d_ = 6.67 × 10^−7^ M), which is about 18-fold weaker than that with L-arginine. Moreover, the PBPb-I domain binds L-ornithine (K_d_ = 4.17 × 10^−6^ M), L-histidine (K_d_ = 2.32 × 10^−5^ M) and L-glutamine (K_d_ = 6.76 × 10^−5^ M) with lower binding affinities, and L-aspartate with no measurable binding affinity. These findings suggested that the CdgH PBPb-I domain exhibits the highest affinity for binding L-arginine, L-lysine, and L-ornithine, but also possesses the ability to bind L-glutamine and L-histidine.

We also tested the interactions between the PBPb-I domain mutants (PBPb-I^E56A^, PBPb-I^R109A^, PBPb-I^E122A^) with different ligands (L-lysine, L-ornithine, L-glutamine, and L-histidine) by ITC (Fig. S3 and Table 2). The ITC results showed that PBPb-I^E122A^ and PBPb-I^R109A^ mutants led to the largest reductions in binding affinity for all these ligands while PBPb-I^E56A^ resulted in smaller but obvious reductions in binding affinity, respectively, relative to the wild-type PBPb-I domain. These results are consistent with the mutation analysis of PBPb-I domain binding to L-arginine, indicating that CdgH PBPb-I domain may employ the same ligand binding pocket to recognize a variety of substrates.

### The potential ligand-binding properties of the PBPb-II domain

**Figure 4:**
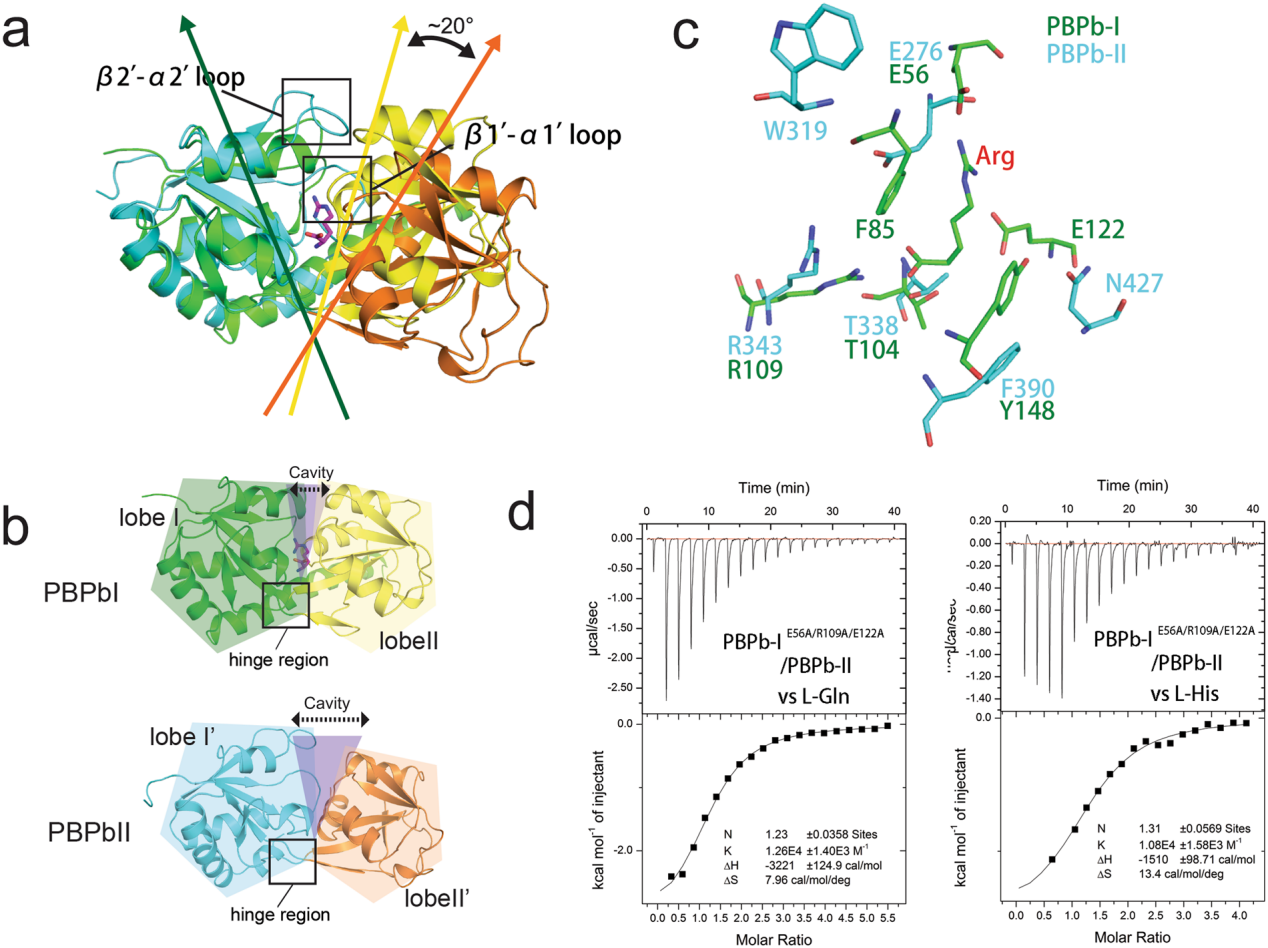
Structural comparison between the PBPb-I and PBPb-II domains. (**a**) Structural superposition of the PBPb-I and PBPb-II domains. The regions affecting the ligand-binding pocket of the PBPb-II domain are shown in the red rectangle. The opening angles for the PBPbs are indicated. Lobe-I and lobe-II of the PBPb-I domain are shown in green and yellow, respectively, while lobe-I′ and lobe-II′ of the PBPb-II domain are shown in cyan and orange, respectively. The L-arginine bound in the ligand-binding pocket is shown in magenta. (**b**) Ribbon representation of the closed PBPb-I and open PBPb-II domains. The cavities are shown in light magenta. (**c**) Superposition of the key ligand-binding residues of the PBPb-II domain (cyan) and PBPb-I domains (green). (**d**) Data from ITC experiments involving PBPb-I^E56A/R109A/E122A^/PBPb-II with L-glutamine and L-histidine.

The CdgH PBPb-II domain of our structure exhibited a ligand-free conformation. Structural superposition of the PBPb-I and -II domains revealed that the L-arginine liganded PBPb-I domain adopts a more closed state compared with the unliganded PBPb-II domain (Fig. 4a). In the PBPb-II domain lacking a bound ligand, the β2′-α2′ loop is located farther away from the ligand-binding pocket and promotes an open conformation, and the β1′-α1′ loop was inserted into the interlobe cavity. Moreover, the flexible hinge regions (β4′-β5′ and β10′-β11′ loops) are situated between lobe-I′ and -II′, leading to localization of lobe-II′ farther away from lobe-I′, thereby enlarging the ligand-binding cavity relative to that observed in the PBPb-I domain (Fig. 4b).

DALI results suggested that the PBPb-II domain might also have the ability to bind a variety of amino acid ligands. Superposition of the ligand-binding pockets of the two PBPb domains revealed that the PBPb-II domain possesses a subset of residues for proposed ligand binding (Fig. 4c). Specifically, the residues E276, T338, and R343 in the PBPb-II domain correspond to E56, T104, and R109 in the PBPb-I domain. Moreover, two aromatic residues (W319 and F390) in PBPb-II correspond to F85 and Y148 in PBPb-I, although W319 located on the β2′-α2′ loop is located farther away from the ligand-binding pocket in the open conformation. Additionally, N427 in the PBPb-II domain correspond to the locations of aspartate residues in other amino acid-binding proteins (D167 of AncQR, D161 of LAOBP, and D157 of GlnBP), but differed from that of E122 in the PBPb-I domain (Fig. 3b and Fig. S1).

To determine the ligand-binding affinity of the PBPb-II domain, we attempted to express this domain exclusive of PBPb-I; however, we found the CdgH PBPb-II domain was insoluble when expressed in *E. coli* alone. Therefore, we created the tandem PBPb domains of CdgH with E56A/R109A/E122A triple mutant (named PBPb-I^E56A/R109A/E122A^/PBPb-II, whose binding ability of the PBPb-I domain is eliminated) to test the ligand-binding properties of the PBPb-II domain by ITC experiments. The results showed that PBPb-I^E56A/R109A/E122A^/PBPb-II binds L-glutamine (K_d_ = 7.94 × 10^−5^ M) and L-histidine (K_d_ = 9.25 × 10^−5^ M) with the highest affinities (Fig. 4d and Table 2), and L-arginine (K_d_ = 1.39 × 10^−3^ M), L-lysine (K_d_ = 2.26 × 10^−3^ M), and L-ornithine (K_d_ = 3.18 × 10^−3^ M) with much lower affinities (Fig. S4 and Table 2), whereas L-aspartate with no measurable binding affinity. These results indicated that the CdgH PBPb-II domain exhibited a greater preference for L-glutamine and L-histidine, with lower affinity for L-arginine, L-lysine, and L-ornithine. However, the ligand-binding affinities are much lower as compared with those measured for the PBPb-I domain. Further studies involving the structures of the ligand-bound PBPb-II domain are needed to confirm PBPb-II domain ligand-binding ability.

### Dimerization of the periplasmic portion of CdgH

Similar to many DGCs, homodimerization leads to alignment of two GGDEF domains, resulting in two-fold symmetry that enables the enzyme to catalyze c-diGMP synthesis^19, 30–32^. A previous report showed that CdgH is capable of interacting with itself^33^, thus we speculated that the periplasmic portion of CdgH might also function as a dimer.

**Figure 5:**
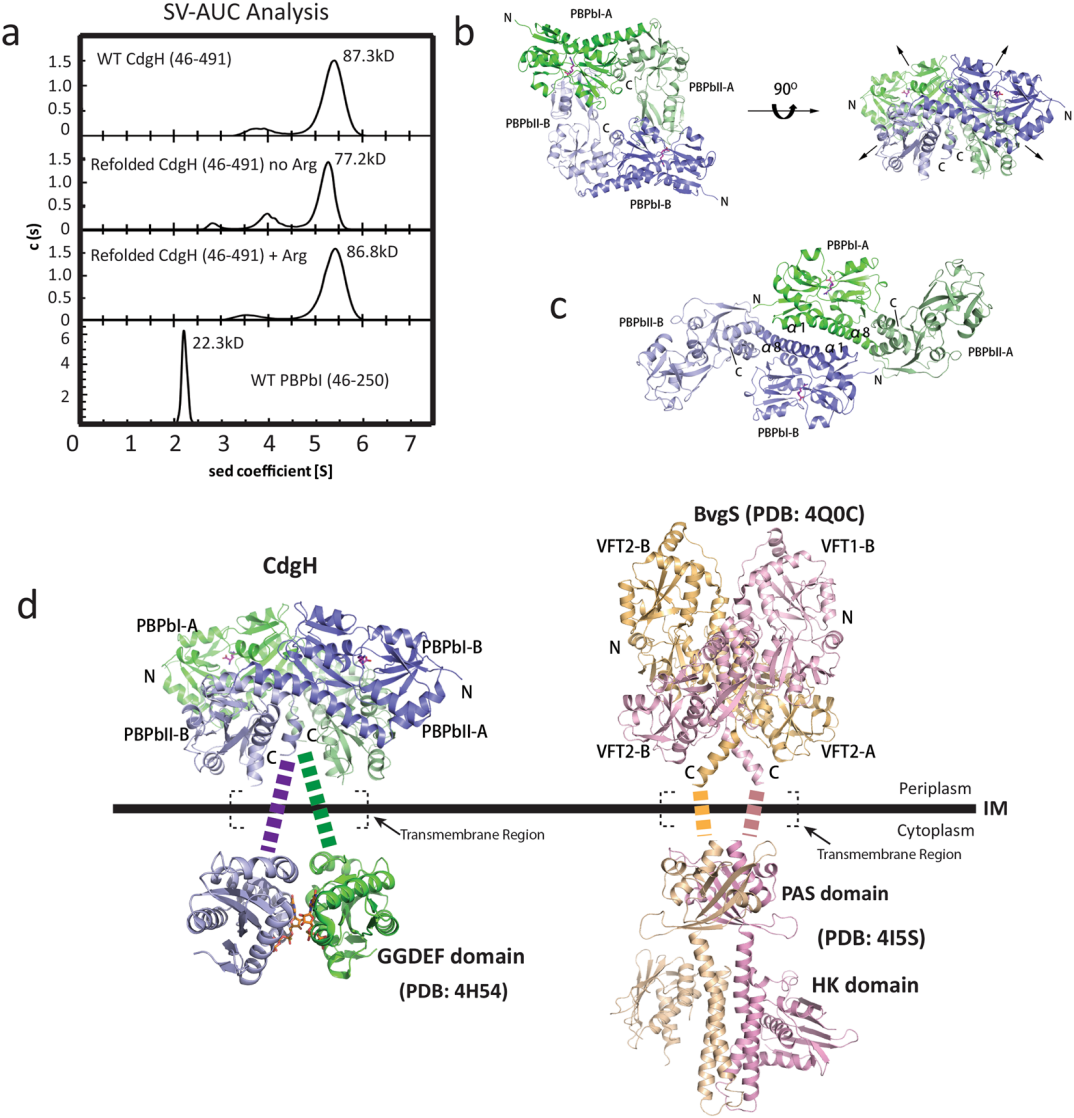
The dimerization of the periplasmic portion of CdgH. (**a**) The SV-AUC results of the wild-type CdgH (46–491), refolded CdgH (46–491), refolded CdgH (46–491) with L-arginine, and the wild-type PBPb-I domain. The calculated molecular mass of the samples are indicated beside the peaks. (**b**) Top view and side view of the type-I dimeric assembly of CdgH (46–491). (**c**) The overall structure of the type-II dimeric assembly of CdgH (46–491). The PBPb-I and -II domains of molecule A are shown in green and light green, respectively, and those of molecule B are shown in blue and light blue, respectively. (**d**) Comparison of the dimeric assemblies of CdgH and BvgS. The cartoon model of the GGDEF domain is generated from the structure of a zinc-sensory diguanylate cyclase from *E. coli* (PDB ID: 4H54). Substrate analog GTPαS molecules are shown as orange sticks. The cartoon model of the tandem periplasmic domains of BvgS is generated from the structure of BvgS (PDB ID: 4Q0C). The cartoon model of PAS (Per-Arnt-Sim) and HK (Histidine Kinase) domains of BvgS is generated from the structure of VicK from *Streptococcus mutans* (PDB ID: 4I5S). The transmembrane regions of both models are indicated by dash lines.

To test this, we first determined the oligomeric state of the unrefolded wild type periplasmic portion of CdgH by analytical ultracentrifugation. The results showed a calculated molecular mass of 87.3 kDa, close to the theoretical molecular weight of the dimer (101.6 kD) (Fig. 5a), suggesting that the periplasmic portion of CdgH exists as a dimer in solution.

Moreover, we analyzed the crystal-packing characteristics of the two protomers in relation to their localization within the crystallographic asymmetric units. Investigation of the neighboring asymmetric units revealed two types of dimeric architecture (Fig. 5b and c), whose buried surface areas are about 1661.6 Å^2^ (type I dimer) and 1039.2 Å^2^ (type II dimer), respectively, and values of CSS (Complex Formation Significance Score, which ranges from 0 to 1 as interface relevance to complex formation increases) are 1 and 0, respectively, suggesting the interface of the type II dimer seems to be a result of crystal packing only. In addition, we analyzed the interfaces of both dimer types. In the type I dimer, the lobe-II′ of the PBPb-II domain of each protomer interacts with the hinge region of the PBPb-I domain of the opposing protomer (Fig. 5b), revealing a dimerization mode requiring both tandem PBPb domains. The type II dimer is formed by the α1 and N-terminal portion of α8 helices packing of the PBPb-I domain of each protomer (Fig. 5c), revealing a dimerization mode requiring only PBPb-I domain. To elucidate which dimer type exists in solution, an analytical ultracentrifugation experiment was also performed for the PBPb-I domain (residues 46–250). The result showed a calculated molecular mass of 22.3 kD (Fig. 5a), close to the theoretical monomer molecular weight (23.1 kD), indicating that the PBPbI domain exists as a monomer and is not enough for dimerization. In addition, we were unable to obtain similar results for the PBPb-II domain alone due to its insolubility in solution. This data, together with structural analysis, indicated that the type II dimer is formed by crystal packing, while the type I dimer is the probable dimer existing in solution and both tandem PBPb domains are required for homodimer formation.

Additionally, in the structure of the type I dimer, both PBPb-I domains are membrane-distal with the pockets oriented relatively upwards, and both PBPb-II domains are membrane-proximal with the pockets relatively downwards. Moreover, the two C-terminal α8′ helices serving as pre-transmembrane sequences at the membrane-proximal end of the structure locate adjacent to each one and can be connected to the subsequent transmembrane helices (Fig. 5b).

### L-arginine induce conformational change of the periplasmic tandem domains of CdgH

To learn more about the dimerization of the periplasmic tandem domains of CdgH, we also performed an analytical ultracentrifugation experiment of the refolded periplasmic portion of CdgH without L-arginine and refolded protein saturated with excess L-arginine. Interestingly, the results showed that the calculated molecular mass of the refolded protein saturated with excess L-arginine is 86.8 kD, nearly the same as that of the unrefolded wild type protein (87.3 kD), is larger than that of the refolded protein without L-arginine (77.2 kD) (Fig. 5a), revealing a more compact state of the liganded dimer than that of the unliganded dimer, suggesting that upon L-arginine binding, the architecture of the periplasmic tandem PBPb domains of CdgH may undergo a conformational changes from loose dimer to compact dimer. This is in line with our structure that L-arginine binding in the pocket will cause the conformational change of the structure from open state to closed state.

Interestingly, BvgS, the only other reported structure containing tandem periplasmic domains found by DALI server, also forms a dimer with a ligand-free state, which is consistent with our analytical ultracentrifugation result that the tandem PBPb domains of CdgH without L-arginine also form a dimer in solution. Despite the differences in the arrangements of the tandem periplasmic domains of CdgH and BvgS, their dimeric architectures both require the entire tandem periplasmic domains, as the PBPb-II/VFT2 domain of each protomer is to interact with the PBPb-I/VFT1 domain of the opposing protomer to form the dimer (Fig. 5d). Moreover, the C-terminal pre-transmembrane helices of the protomers both locate in the center position in both CdgH’s and BvgS’s dimeric architectures. This feature makes it possible that the clamshell motions of the tandem domains upon ligand-binding lead to the conformational and dynamic changes of the transmembrane and cytoplasmic portions, thus affect the downstream DGC activity of the CdgH. However, more studies are needed to confirm this hypothesis.

## Discussion

C-di-GMP is a second messenger in bacterial signal transduction. Most DGCs contain conserved C-terminal GGDEF domains enabling synthesis of c-di-GMP, as well as highly variable N-terminal signal-recognition domains for responding to specific environmental signals, such as PAS (Per-Arnt-Sim), GAF (cGMP phosphodiesterase/adenylate cyclase/FhlA transcriptional activator), REC (receiver) domains and so on^5, 15, 20, 34^. However, little is known about the detailed mechanisms associated with how these DGC domains activate the C-terminal GGDEF domains in response to environmental signals to initiate subsequent signal transduction pathways. There are 31 DGC proteins found in *V. cholera*; however, to our knowledge, no structure of *V. cholerae* DGCs has been reported. Sequence analysis indicated that the periplasmic portion of the CdgH DGC employs two tandem PBPb domains^21^, but the function of the periplasmic portion of CgdH remains elusive.

Here, we solved the crystal structure of the periplasmic portion of CdgH containing tandem PBPb-I and -II domains. To our knowledge, this is the first structure of the periplasmic tandem PBPb domains of a *V. cholera* DGC. Unexpectedly, our structure shows that the PBPb-I domain is in complex with L-arginine which may be co-purified from the *E. coli* expression system, whereas the PBPb-II domain shows no ligand bound. Furthermore, our results indicated that the PBPb-I domain binds various amino acids, with a higher affinity for L-arginine, L-lysine, and L-ornithine, whereas the PBPb-II domain exhibited higher affinities for L-glutamine and L-histidine.

Structural analysis revealed that the binding of L-arginine resulted in a conformational change in the PBPb-I domain from open to closed. We also observed dimerization of the periplasmic portion of CdgH both in solution and in the crystal-packing alignment. Moreover, both calculated molecular masses of the unrefolded wild type protein (87.3 kD) and refolded protein with excess L-arginine (86.8 kD) are larger than that of the refolded protein without L-arginine (77.2 kD), revealing that the liganded dimer is more compact than the unliganded dimer upon L-arginine binding.

Meanwhile, it has been reported that amino acids, including L-arginine, can modulate biofilm formation and swarming motility of *P. aeruginosa* by controlling the intracellular levels of c-di-GMP^35^. In addition, *S*. Typhimurium was shown to specifically respond to L-arginine with an increase in c-di-GMP, and the responses required the regulating periplasmic domain of the diguanylate cyclase STM1987^36^. Together with these previous reports, our structural and functional studies suggest that L-arginine might also function as a small molecule modulator for the DGC activity of CdgH. Under low concentrations of L-arginine in environmental conditions, the periplasmic portion of CdgH exhibits as a loose dimer with both open apo conformations of the PBPb-I and -II domains. Upon L-arginine binding, the dimer undergoes conformational changes from the loose state to the compact state, subsequently causes rotation and translocation of the transmembrane helices, thereby enables rearrangement of the C-terminal GGDEF domains and affects subsequent enzyme activation.

The novel ligand-binding mode of the periplasmic portion of CdgH illustrated here provided new insights into the selectivity for various amino acids among different ligand-binding proteins. Moreover, this work revealed the first structure of a tandem PBPb-containing domain related to rugosity and biofilm formation in *V. cholerae* and may facilitate the development and optimization of anti-biofilm drugs for the treatment of infections.

## Methods

### Protein expression

The periplasmic portion (residues 46–491), the PBPb-I domain (residues 46–250) and the PBPb-II domain (residues 251–491) of *V. cholerae* CdgH were cloned into the pET22b vector (Merck Millipore, Darmstadt, Germany) via the *Nde*I and *Xho*I restriction sites, with a constructed C-terminal His_6_, respectively. Site-directed mutagenesis was performed using a QuikChange kit (Agilent Technologies, Santa Clara, CA, USA) according to manufacturer instructions.

The proteins were overexpressed by the *E. coli* BL21 (DE3)-CodonPlus-RIPL strain in LB medium. Protein expression was induced by adding 0.5 mM isopropyl β-D-1-thiogalactopyranoside at an OD_600_ of ~0.8, followed by incubation of the cell cultures overnight at 16 °C. The cells were subsequently harvested by centrifugation and stored at −80 °C.

### Protein purification

Cell suspensions were thawed, resuspended in binding buffer (20 mM Tris, 500 mM NaCl, 20 mM imidazole [pH 8.0]), and homogenized using a high-pressure homogenizer (JNBIO, Beijing, China). After centrifugation by 16,000 rpm for 30 min at 4 °C, the supernatant was loaded onto a 5-ml Ni-affinity column (GE Healthcare) equilibrated with binding buffer.

For ITC experiments, except for the PBPb-I^E56A/R109A/E122A^/PBPb-II mutant that is purified without refolding, other proteins were first purified by Ni-affinity chromatography with on-column refolding which ensured complete removal of endogenously bound ligands. The Ni-affinity column pre-loaded with the proteins was washed with 50 ml binding buffer followed by 50 ml unfolding buffer (8 M urea, 20 mM Tris, 500 mM NaCl [pH 8.0]). Proteins were refolded on-column by application of a gradient from unfolding buffer to binding buffer over 100 min at a flow rate of 2 ml/min using an FPLC (GE Healthcare), then eluted with elution buffer (20 mM Tris, 500 mM NaCl, 500 mM imidazole [pH 8.0]). Then the refolded proteins were exchanged into buffer A (20 mM Tris, 50 mM NaCl [pH 8.0]), loaded onto an anion-exchange column (Resource Q; GE Healthcare) equilibrated with buffer A and eluted by a gradient from buffer A to buffer B (20 mM Tris, 1 M NaCl [pH 8.0]). Finally, the proteins were purified by a size-exclusion column (Superdex 200; GE Healthcare) in Tris buffer (20 mM Tris, 150 mM NaCl [pH 8.0]).

The periplasmic portion of CdgH for crystallization and the PBPb-I^E56A/R109A/E122A^/PBPb-II mutant for ITC assays were purified by Ni-affinity chromatography without refolding, followed by an anion-exchange column (Resource Q; GE Healthcare) and a size-exclusion column (Superdex 200; GE Healthcare).

All of the purified fractions were collected and concentrated to ~40 mg/mL in Tris buffer (20 mM Tris, 150 mM NaCl [pH 8.0]), followed by freezing in liquid nitrogen and storage at −80 °C.

### Crystallization and data collection

The purified periplasmic portion of CdgH without refolding was used for crystallization. Crystal screening was performed with commercial screening kits (Hampton Research, Aliso Viejo, CA, USA) using the sitting-drop vapor diffusion method, and positive hits were optimized using the hanging drop vapor diffusion method at 293 K. SeMet crystals of the periplasmic portion of CdgH protein were obtained and optimized in buffer containing 0.1 M Tris-HCl (pH 8.0) and 10% w/v polyethylene glycol 8000 using the concentration of 10 mg/ml. After soaking for 3 min in cryoprotection solution (well solution complemented with 15% sucrose), the crystals were cooled by plunging them into liquid nitrogen. A single diffraction dataset (360 images with an oscillating range of 1°) of the best crystal (diffracting at 2.60 Å) was collected at a wavelength of 0.97848 Å and a temperature of 100 K on beamline BL18U at the Shanghai Synchrotron Radiation Facility (SSRF).

### Structure determination and refinement

Diffraction images were indexed and scaled using the HKL2000 software program^37^. The crystal space group is P6_3_22, with one molecule in the asymmetric unit. The phase was determined using single-wavelength anomalous diffraction (SAD) method based on the selenomethionine-substituted protein crystal. Electron-density maps were calculated using PHENIX^38^, and model building was performed using COOT^39^ and refined using PHENIX. refine^38, 40^. The final structures were analyzed with PROCHECK^41^. The structure was refined to final R_work_ of 22.92% and R_free_ of 28.85%. A Ramachandran analysis indicated that 91% of residues are in most favored region, 7% in additionally allowed region and 1.9% in disallowed region. Data collection and refinement statistics are presented in Table 1, and the figures depicting structures were prepared using PyMOL (http://www.pymol.org). Atomic coordinates and structure factors have been deposited in the RCSB Protein Data Bank (http://www.pdb.org) under accession codes 5 GZS.

### ITC assays

For ITC experiments, except for PBPb-I^E56A/R109A/E122A^/PBPb-II whose binding ability of the PBPb-I domain is eliminated, other proteins were all refolded and purified as described above. ITC experiments were performed in a buffer composed of 20 mM Tris (pH 8.0) and 150 mM NaCl at 25 °C using VP-ITC or iTC200 calorimeters (GE Healthcare). For VP-ITC calorimetry experiments, L-Arg (0.08 mM) was titrated into the tandem PBPb domains (0.02 mM), and the titration sequence included a single 2 µl injection, followed by 55 injections of 5 µl each, with a 210 s interval between injections and a stirring rate of 307 rpm. In addition, various amino acids (0.5 mM) were titrated into the PBPb-I domain and its mutants (0.05 mM), respectively, and the titration sequence included a single 2 µl injection, followed by 27 injections of 10 µl each, with a 210 s interval between injections and a stirring rate of 307 rpm. For iTC200 calorimetry experiments, various amino acids (8 mM) were titrated into the PBPb-I^E56A/R109A/E122A^/PBPb-II protein (0.2 mM), and the titration sequence included a single 0.5 µL injection, followed by 19 injections of 2 µL each, with a 2 min interval between injection and a stirring rate of 1000 rpm. Each ITC experiment was performed twice. Calorimetric data were analyzed using OriginLab software (GE Healthcare). For titrations with *c* > 1, where *c* is the product of the association constant (Ka) for the interaction and the protein concentration in the cell, the stoichiometry (n), enthalpy (ΔH), and Ka for the interaction were determined. For titrations with *c* < 1, n was fixed at 1 while ΔH and Ka were determined. The ITC data are presented in Table 2.

### Analytical ultracentrifugation

Sedimentation velocity measurements (SV-AUC) were performed on a Beckman ProteomeLab XL-I (Beckman Coulter, Brea, CA, USA) at 25 °C. All protein samples were diluted to a concentration of 0.7 mg/ml in 20 mM Tris (pH 8.0) and 150 mM NaCl. The data of PBPb-I domain were collected at 60,000 rpm, while the tandem PBPb domains unrefolded and refolded with or without excess L-arginine at 40,000 rpm every 3 min at a wavelength of 280 nm. Interference sedimentation coefficient distributions were calculated from the sedimentation velocity data using SEDFIT^42^.

## Electronic supplementary material

supplementary data
