# Supplementary material for: Structural studies of the periplasmic portion of the diguanylate cyclase CdgH from Vibrio cholerae
Source: Sci Rep. 2017 May 12;7:1861. doi: 10.1038/s41598-017-01989-6 (PMC5431781; doi:10.1038/s41598-017-01989-6)
Supplement: Supplementary file 1 — supplementary data [file 41598_2017_1989_MOESM1_ESM.doc]

**Structural studies of the periplasmic portion of the diguanylate cyclase CdgH from *Vibrio cholerae***

Min Xu^1,+,*^, Yi-Zhi Wang^1,2,+^, Xiu-An Yang^1,+^, Tao Jiang^1,2^, Wei Xie^3,*^

1. National Laboratory of Biomacromolecules, CAS Center for Excellence in Biomacromolecules, Institute of Biophysics, Chinese Academy of Sciences, Beijing, China
2. University of Chinese Academy of Sciences, Beijing, China
3. School of life sciences, Tianjin University, Tianjin, China

+ co-first author

* corresponding author

Min Xu < xumin@moon.ibp.ac.cn >;

Wei Xie <xiewei@tju.edu.cn>.

**
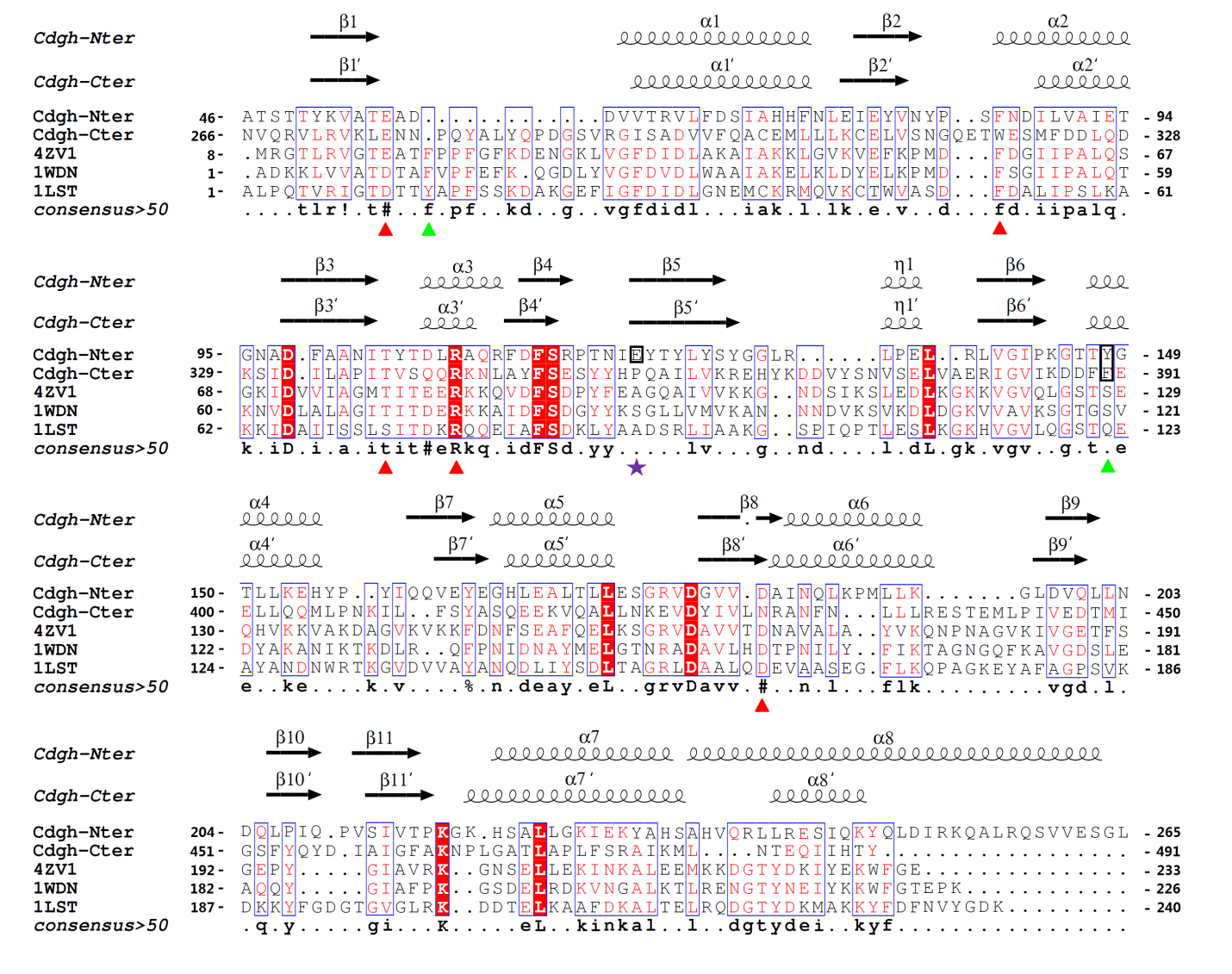
**

**Figure S1. The sequence alignment of the PBPb domains of CdgH and other structural homology proteins.** The α-helices and β-sheets of PBPb-I (CdgH-Nter) and PBPb-II (CdgH-Cter) domains are marked with black lines. In the alignment, conserved residues are white; similar residues are red; variable residues are black. Red triangles, conserved residues in the ligand-binding pockets; green triangles, the unique hydrophobic residues (Y148 and W319 from CdgH) in the ligand-binding pockets of CdgH PBPb domains compared with other ligand-binding proteins (F20 of 4ZVI, Y14 of 1LST, and F13 of 1WDN); Magenta star, E122 residue of CdgH PBPb-I domain.


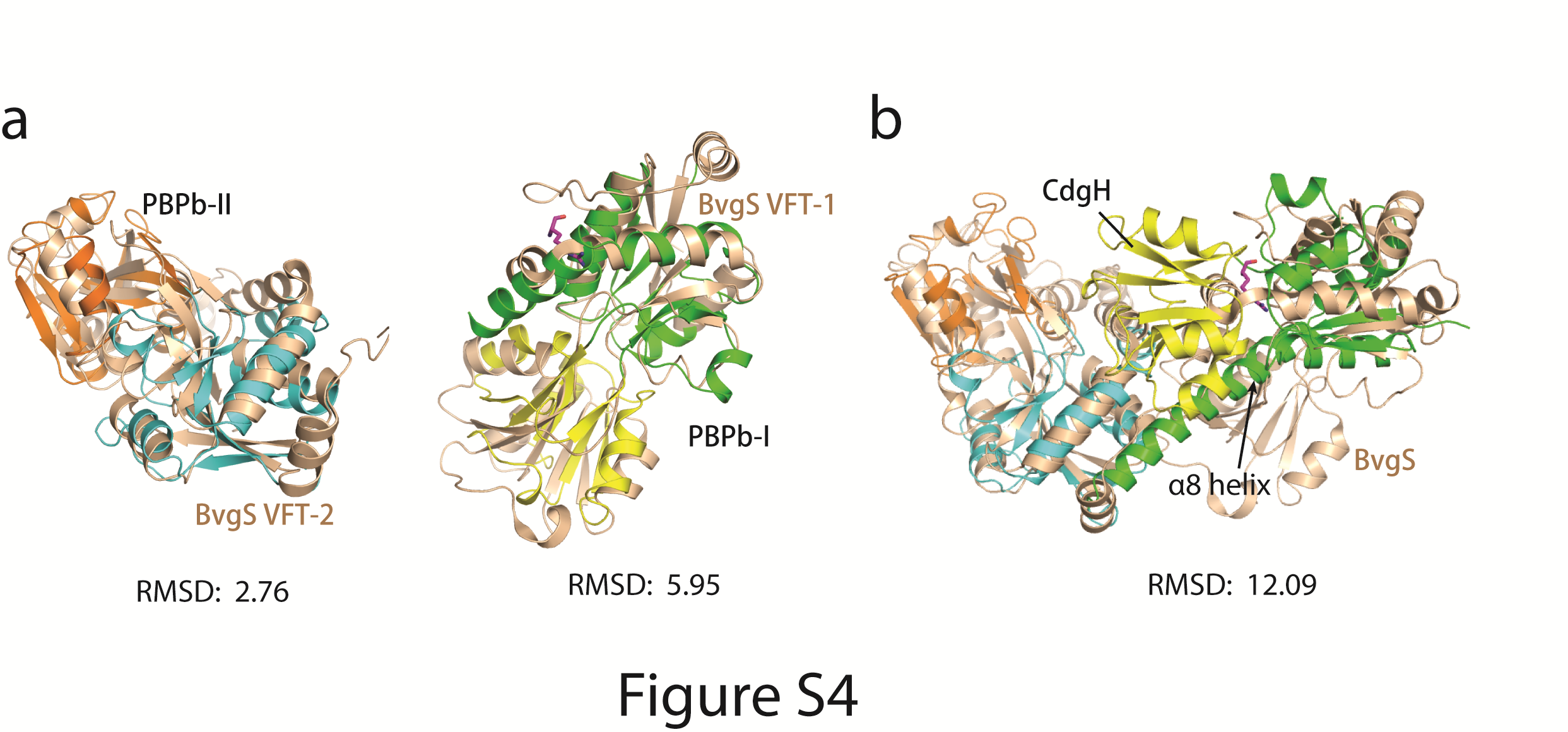


**Figure S2.** Structural comparison between the periplasmic portions of CdgH and BvgS. (a) Structural superposition of the PBPbI/VFT1 and PBPb-II/VFT2 domains. (b) Structural superposition of the overall structures of the periplasmic portions of CdgH and BvgS. The values of root mean square deviation (RMSD) are indicated. Lobe-I and lobe-II of the PBPb-I domain are shown in green and yellow, respectively, while lobe-I′and lobe-II′ of the PBPb-II domain are shown in cyan and orange, respectively. The VFT1 and VFT2 domains of BvgS are shown in wheat.


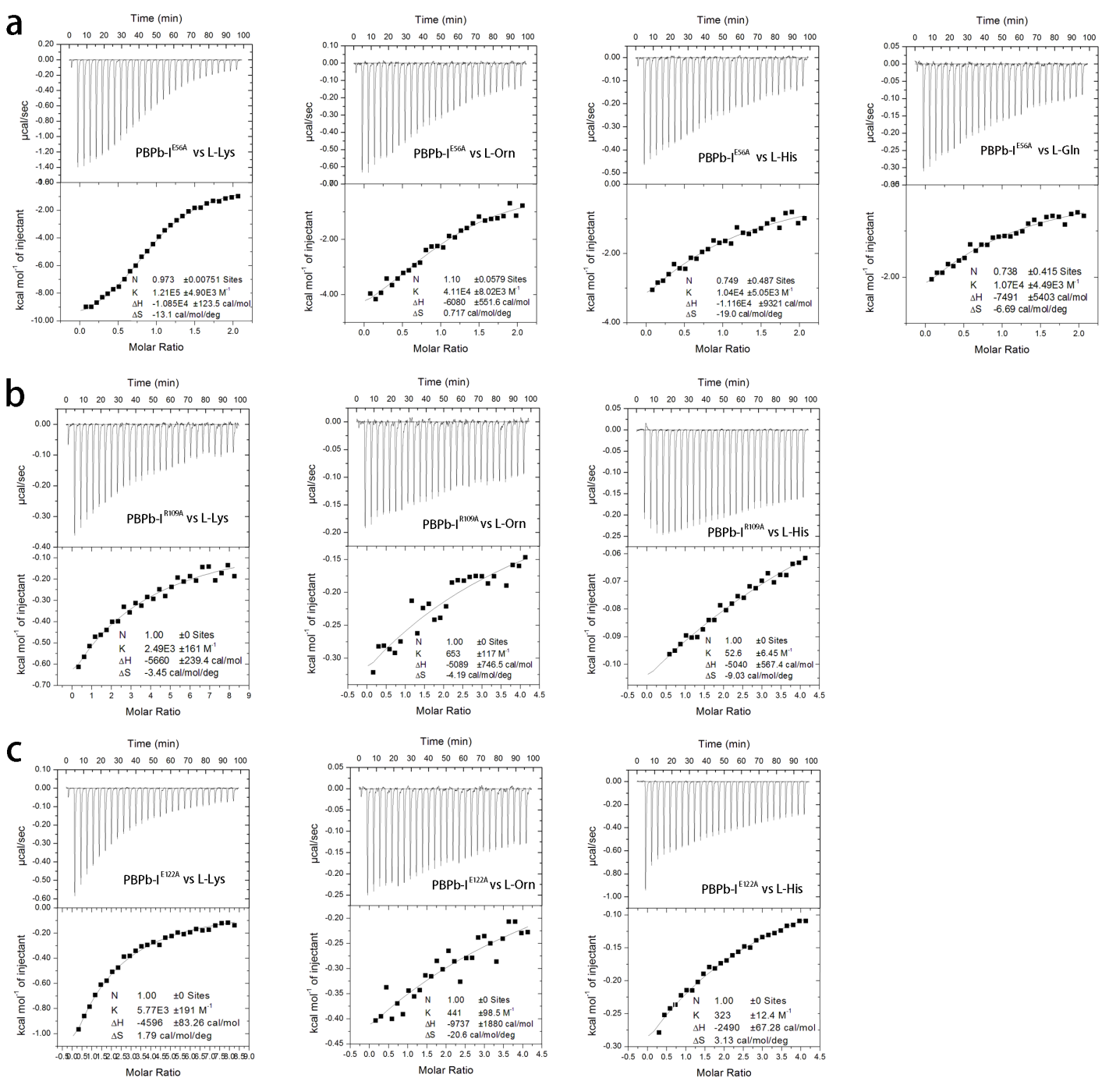


**Figure S3.** Data from ITC experiments involving the PBPb-I mutants (PBPb-I^E56A^, PBPb-I^R109A^, PBPb-I^E122A^) with different ligands (L-lysine, L-ornithine, L-histidine and L-glutamine).


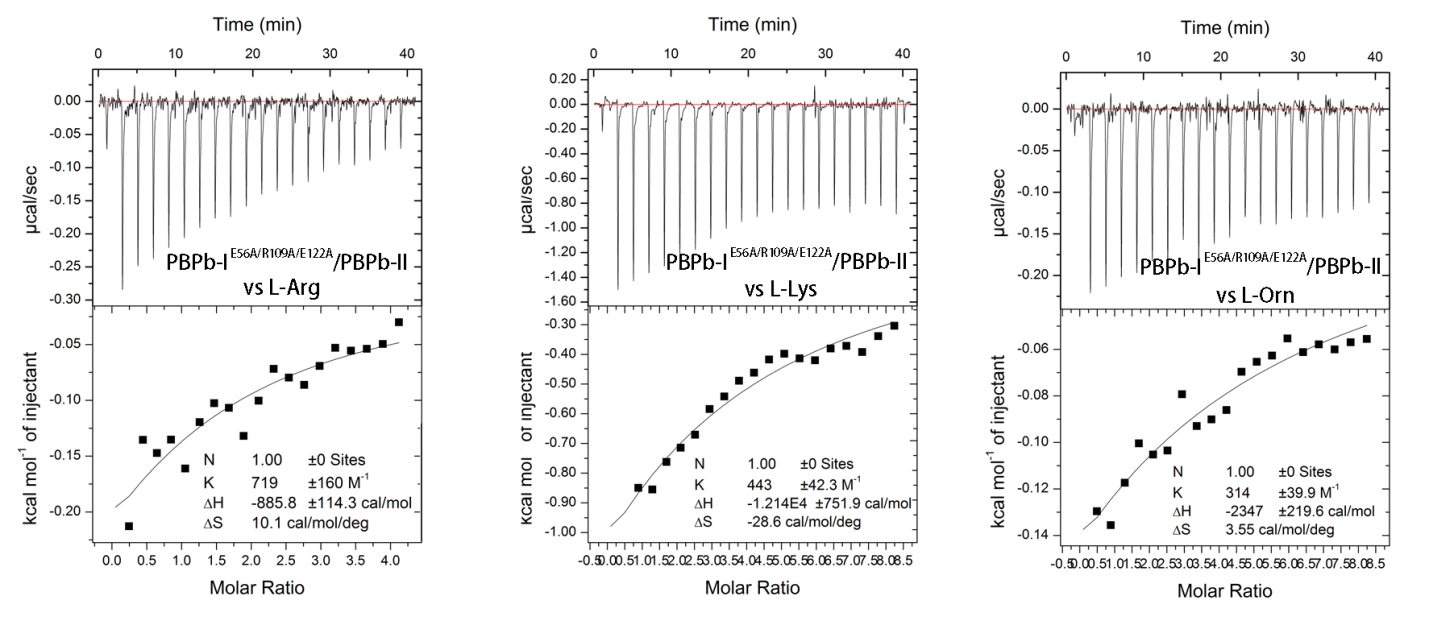


**Figure S4.** Data from ITC experiments involving PBPb-I^E56A/R109A/E122A^/PBPb-II with different ligands (L-arginine, L-lysine, and L-ornithine).
